# *Biomphalaria glabrata* transcriptome: Identification of cell-signalling, transcriptional control and immune-related genes from open reading frame expressed sequence tags (ORESTES)

**DOI:** 10.1016/j.dci.2006.11.004

**Published:** 2007

**Authors:** Anne E. Lockyer, Jennifer N. Spinks, Anthony J. Walker, Richard A. Kane, Leslie R. Noble, David Rollinson, Emmanuel Dias-Neto, Catherine S. Jones

**Affiliations:** a Wolfson Wellcome Biomedical Laboratory, The Natural History Museum, Cromwell Road, London SW7 5BD, UK; b School of Life Sciences, Kingston University, Penrhyn Road, Kingston upon Thames, Surrey KT1 2EE, UK; c School of Biological Sciences, Aberdeen University, Tillydrone Avenue, Aberdeen AB24 2TZ, UK; d Laboratorio de Neurociencias (LIM27), Instituto de Psiquiatria, Faculdade de Medicina da Universidade de Sao Paulo, Sao Paulo, SP, Brazil; e MD Anderson Cancer Center, GU Medical Oncology Department, University of Texas, 1515 Holcombe Blvd, Unit 1374, Medical Center, Houston, TX 77030, USA

**Keywords:** Expressed sequence tag, EST, ORESTES, Molluscan defence, *Biomphalaria glabrata*, *Schistosoma mansoni*

## Abstract

*Biomphalaria glabrata* is the major intermediate snail host for *Schistosoma mansoni*, one of the important schistosomes infecting man. Much remains to be discovered concerning specific molecules mediating the defence events in these intermediate hosts, triggered by invading schistosomes. An expressed sequence tag (EST) gene discovery strategy known as ORESTES has been employed to identify transcripts that might be involved in snail–schistosome interactions in order to examine gene expression patterns in infected *B. glabrata*. Over 3930 ESTs were sequenced from cDNA libraries made from both schistosome-exposed and unexposed snails using different tissue types, producing a database of 1843 non-redundant clones. The non-redundant set has been assessed for gene ontology and KEGG pathway assignments. This approach has revealed a number of signalling, antioxidant and immune-related gene homologues that, based on current understanding of molluscan and other comparative systems, might play an important role in the molluscan defence response towards infection.

## 1 Introduction

The freshwater snail *Biomphalaria glabrata* is an intermediate host for *Schistosoma mansoni*, the digenean parasite that causes human intestinal schistosomiasis. This host–parasite relationship has become a model system for examination of snail–schistosome interactions, and as such, recent molecular work has focused on *B. glabrata*. Now with the continued significance of genome research, the *B. glabrata* genome initiative (http://biology.unm.edu/biomphalaria-genome/) aims to increase the available genetic data for this snail species, with the final goal of a complete genome sequence. Such sequence data will complement that available for the schistosome parasite from the schistosome genome/transcriptome sequencing initiatives [1–5] and for the definitive host from the human genome project [6]. In addition to genome sequencing, the generation of expressed sequence tags (ESTs), short stretches of sequence obtained from cDNA libraries [7], is valuable in a number of ways: in identifying snail homologues of genes previously described in other species; for identifying transcribed regions of the genome, useful for genome annotation and analysis; for the detection of splice variants and alternative polyadenylation gene isoforms; in the discovery of single nucleotide polymorphisms (SNPs) and finally for expression studies, such as those involving microarrays. The EST project described here was initiated with the ultimate aim of manufacturing a cDNA microarray for *B. glabrata,* which required a large number of sequenced cDNA clones to be available.

EST projects in other molluscs, such as oysters, have revealed a wealth of useful sequence data including signalling, antioxidant and immune-related gene homologues [8,9], demonstrating that molluscs express many of the same genes, and may therefore carry out the same processes, which have previously been described in vertebrates. A recent EST project from *Lymnaea stagnalis* [10] identified a number of genes that had not previously been identified in the Lophotrochozoa. Therefore initiating an EST sequencing project in *B. glabrata* has the potential to identify other novel molluscan genes including those that might be associated with the snail's response to infection. At the start of this project (January 2003) only 1427 *B. glabrata* EST sequences were available on GenBank from earlier studies [11–15]. During the course of this project several other laboratories have also developed gene discovery programmes for *B. glabrata* [16,17] (see also http://biology.unm.edu/biomphalaria-genome/detailing unpublished EST programmes).

Previous EST projects in *B. glabrata* [11,15] used traditional library construction and sequencing approaches to obtain sequence data. A complimentary EST approach called open reading frame ESTs (ORESTES) [18] has been used successfully to obtain large numbers of sequences for both human [18–20] and schistosome [4,5] transcriptome projects. The ORESTES approach preferentially targets the middle section of mRNAs [18], making it more likely coding regions will be sequenced, than in other EST methodologies where sequencing commences at the end of the cDNA, often only obtaining untranslated sequence. This alternative method has two advantages for snail ESTs; firstly, it is more likely that gene similarity to other organisms can be ascertained if coding regions are sequenced, and secondly, the data generated are likely to be complementary to, rather than redundant with, sequence data from traditional approaches. The ORESTES approach also allows the construction of a number of mini-libraries using small quantities of RNA [21], making it suitable for investigating gene expression in small amounts of tissue such as those present in *B. glabrata*. Producing a large number of smaller libraries also facilitates a more extensive analysis of gene expression; thus in the EST project described here, different snail strains (both resistant and susceptible to *S. mansoni* infection) were used and different tissue types from both parasite-exposed and unexposed material were examined. Based on gene ontology and Kyoto Encyclopaedia of Genes and Genomes (KEGG) pathway assignments a number of antioxidant, signalling and immune-related gene homologues have been identified and are presented here; the potential involvement of these genes in molluscan defence is considered, particularly within the framework of comparative immunobiology.

## 2 Materials and methods

### 2.1 Snail material

*B. glabrata* strains used were: resistant BS90 [22] (NHM3017) or susceptible NHM1742 or BB02 (NHM3032), the strain currently being used for the genome sequencing project (see http://biology.unm.edu/biomphalaria-genome/BB02STRAIN.html). Snails were held overnight in autoclaved snail water with 100 μg/ml ampicillin prior to killing by decapitation. The exuded haemolymph was collected, pooled and the haemocytes isolated by centrifugation at 4 °C, 10,000*g* for 20 min. Each snail was preserved in 800 μl RNAlater (Ambion Inc., Texas, USA) and stored at −20 °C until dissection. Haemopoietic organ, ovotestis, head/foot and brain tissue was dissected. For the exposed material, 60 snails were individually exposed to 10 *S. mansoni* miracidia (Belo Horizonte strain) each and 2, 4, 6, 8 and 24 h after infection, 12 of the snails were swiftly killed as above. Tissue was pooled from each time period; the extended sampling was designed to include all transcripts expressed over the first 24 h of infection.

### 2.2 RNA extraction

Total RNA was extracted from each dissected tissue using SV RNA extraction kit (Promega UK Ltd, Southampton, UK) according to the manufacturer's protocol. This kit includes DNAse treatment to eliminate genomic DNA contamination. Pigment from the head/foot tissue was found to block the spin columns supplied with this extraction kit, so RNA was extracted from this tissue using Trizol (Invitrogen Ltd, Paisley, UK). Briefly, 30 mg tissue was ground in 1 ml Trizol and centrifuged at 12,000*g* for 10 min at 4 °C. The supernatant was incubated at room temperature for 5 min then 0.2 ml chloroform added, mixed vigorously and left at room temperature for 3 min. The samples were spun at 12,000*g*, 4 °C for 15 min and the RNA precipitated from the supernatant using 0.5 ml propan-2-ol and centrifugation at 12,000*g* for 10 min at 4 °C. The pellet was washed using 75% ethanol and dissolved in 50–100 μl water. RNA extracted using Trizol was DNAse treated (Promega), according to the manufacturer's instructions prior to mRNA extraction. mRNA was extracted from the total RNA from both extraction methods using the Micro-fastTrack 2.0 mRNA extraction kit (Invitrogen) according to the manufacturer's instructions. The mRNA was eluted in 200 μl elution buffer and precipitated overnight at −70 °C using 600 μl ethanol. The mRNA was dissolved in 10 μl water and tested using specific *B. glabrata* actin primers [12] to check there was no DNA contamination.

### 2.3 cDNA synthesis and amplification

For each library, a 27 μl mastermix containing 800 U Reverse Transcriptase (MMLV-RT) (Promega), 4 μl RNAsin (Promega), 4 μl dNTPs at 2 mM, 8 μl 5× buffer (Promega) and 7 μl mRNA (70–240 ng) was prepared and 2 μl aliquoted into 12 tubes prepared with 12 different arbitrary primers (1.5 μl of 15 mM) (for primer sequences see supplementary material). The tubes were incubated at 42 °C for 1 h then heated to 70 °C for 10 min.

Amplification was carried out using Ready-to-go beads (Amersham Biosience, Amersham, UK). The 3.5 μl cDNA reactions (including primers) and 25 μl water were each added to a tube containing a single bead and amplified using the following cycling conditions: 75 °C for 5 min followed by 15 cycles at 94 °C, 52–45 °C for 1 min (touchdown PCR, dropping 0.5 °C each cycle) and 1 min 72 °C, then 26 cycles of 94 °C for 30 s, 48 °C for 1 min and 72 °C for 1 min, then 7 min at 72 °C. A negative control (no DNA) was carried out simultaneously for each primer (dissolving 2 ready-to-go beads in 50 μl water and aliquoting 3 μl into a tube containing 0.3 μl primer (at 15 mM)). Three μl of each synthesis reaction was examined by gel electrophoresis alongside the control amplification and reactions chosen for inclusion in the mini-library only if the control amplification showed no contamination and a smear without single prominent bands in the reaction profile. This ensured that a mix of products would be obtained from each library.

### 2.4 Cloning and sequencing

For each library, the selected amplified cDNA samples were pooled and cloned using pGEM-T easy cloning kit (Promega) according to the manufacturer's instructions. One hundred and ninety two clones (2×96), selected at random from the cloning plates were picked into 0.5 ml LB and grown up overnight. Ten μl PCRs with M13 forward and reverse primers were carried out to check insert size and the presence of a single insert and, from these, 96 colonies were chosen for 100 μl PCRs. PCRs contained 1×NH_4_ reaction buffer (Bioline, London, UK), 2.5 mM MgCl_2_, 0.2 mM dNTP, 0.2 μM each M13 Forward and Reverse primers and 0.025 U/μl PCR *Taq* polymerase (Bioline, London, UK). Cycling conditions were: 94 °C for 2 min, then 35 cycles of 94 °C for 30 s, 58 °C for 30 s and 72 °C for 1 min 30 s, then 10 min at 72 °C. Glycerol stocks for the selected colonies were stored at −80 °C.

PCR products were purified using Multiscreen PCR filter plates (Millipore, Billerica, USA) then cycle-sequenced directly using BigDye kit (Applied Biosystems, Foster City, USA) and T7 primer and run on ABI 377 or capillary sequencers. Vector, primer and poor quality sequences were removed using Sequencher 3.1.1 (GeneCodes Corp., Ann Arbor, USA).

### 2.5 Bioinformatics

Cluster analysis was performed in SeqTools (http://www.seqtools.dk/) using BlastN score values (cut-off value 0.5) and used to calculate percentage redundancy. For each library BlastN and BlastX [23] searches were run and any ribosomal sequences and sequences shorter then 80 bp removed. Duplicate sequences were also removed, although overlapping sequences were retained. Since each library represented different tissues, strains or infected/uninfected material, duplicate sequences between libraries were retained.

### 2.6 Clone nomenclature

Each clone had a unique ZB number assigned during sequencing. In addition to this the clones were also assigned a code based on strain (see Section 2.1), tissue type (B—brain, HO—haemopoietic organ, HAEM—haemocytes, HF—head/foot and OT—ovotestis), infection status (EX—parasite exposed, UN—unexposed) and plate number and position. The sequences were submitted to GenBank (accession numbers CK149151-CK149590, CK656591-CK656938, CO870183-CO870449, CV548035-CV548805, EG030731-EG030747).

### 2.7 Gene function

Gene ontology functions were assigned using GOblet (http://goblet.molgen.mpg.de/). KEGG pathway analysis was carried out using the KEGG automatic annotation server (KAAS) for ortholog assignment and pathway mapping (http://www.genome.jp/kegg/kaas/).

## 3 Results

### 3.1 ORESTES libraries

A total of 41 ORESTES libraries were made from five tissue types (head–foot, brain, ovotestis, haemocytes and haemopoietic organ) from the three *B. glabrata* strains, one resistant (NHM3017) and two susceptible (NHM1742 and BB02 (NHM3032)), using material that had either remained unexposed or had been exposed to *S. mansoni* (Table 1). Some of the libraries were made from the same snail strain and tissue type but were separately made with different primers, using new experimental material, so have been treated independently. Libraries were prepared from each of the two susceptible strains for each tissue, with the exception of exposed ovotestis from NHM1742 where two libraries were made. Two or three libraries were prepared per tissue and exposure type for the resistant strain, with the exception of brain tissue where material was limiting. A single plate of 96 clones was sequenced for each library and in total, 3936 clones were sequenced from 41 libraries.

### 3.2 Analysis of total ESTs and selection of non-redundant ESTs

A total of 3809 sequences were obtained (127 reactions did not work or the sequenced clones contained no insert or had mixed sequences so these were not analysed further) and were compared to the non-redundant section of GenBank. The Blast results (Table 2) showed that 28.5% (n=1087) of the gene fragments identified proteins on the database, including 35 previously characterized *B. glabrata* proteins and 127 proteins with no assigned function. Including some other non-coding gene fragment matches, less than 2% (n=76) of the sequences matched characterized *B. glabrata* genes or proteins in the non-redundant section of GenBank. Nearly 35% (n=1317) could not be assigned any function, either having no Blast matches, or having homology to a nucleotide or protein sequence on GenBank with no function described. Unfortunately, 39% of the sequences (n=1482) matched *B. glabrata* ribosomal sequences. Other workers [16] have also found a large ribosomal content in polyA selected RNA from *B. glabrata*, and concluded that the high A content in *B. glabrata* ribosomal sequences meant that polyA selection did not efficiently remove it. In the present study it was found that some 18mer primers chosen for ORESTES library construction tended to target ribosomal regions, so they were not used again. However, it was impossible to predict in advance which primers would be problematic. The ‘other’ sequences (n=8) had database matches but do not necessarily code for proteins, for example retrotransposon sequences. For each library, duplicate and ribosomal sequences were removed and the % redundancy per plate ranged from 9.6% to 71.9% (Table 1).

**Fig. 1:**
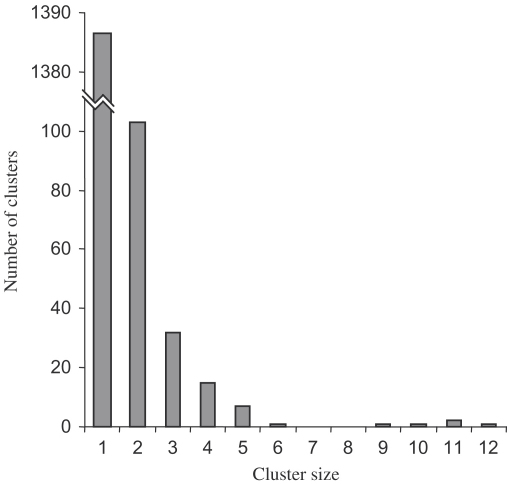
Histogram showing EST clusters in the non-redundant EST set, after removal of duplicates *within* libraries.

After removal of duplicate and ribosomal sequences, a total of 1843 non-redundant sequences were submitted to GenBank, ranging in size from 80 bp (shorter sequences were discarded) to 1068 bp, with a mean length of 518 bp. Cluster analysis between libraries (since the data were previously sifted to remove duplicate clones from each library) revealed 456 sequences in 163 clusters and 1387 singletons (Fig. 1). This resulted in 1550 unique sequences, with 15.9% redundancy. The most common sequence (in 12 of the libraries) was tropomyosin 2 (SwissProt accession number P43689), previously sequenced from *B. glabrata* [24], while two other common sequences (in 11 libraries) were a hypothetical integral membrane transporter protein (accession number XP_135742) and a sequence with no Blast match. Examining the Blast results from the 1843 non-redundant sequences (Table 2), 42% showed significant BlastX similarity to known proteins (including mitochondrial, ribosomal and *Biomphalaria* proteins) in the non-redundant databases, while 52.9% were of unknown function, 3.4% *Biomphalaria* sequences, 0.4% ‘other’ sequences (e.g. retrotransposons) and the remainder were ribosomal sequences (2.4%).

### 3.3 Cluster analysis with other B. glabrata ESTs

Sequences from the 1843 ORESTES clones were used for BlastN searches of the other 10,791 *B. glabrata* EST sequences available on dbEST (September 2006) including many added since the sequences presented here. Four hundred and thirty-nine of the ESTs identified *B. glabrata* sequences with a match greater than 1e-20. Cluster analysis of these revealed 293 clusters or unique ESTs matched sequences on dbEST. Closer examination of a subset of 1613 sequences from a *B. glabrata* haemocyte cDNA library [17], created in the conventional way (not from ORESTES mini-libraries) showed only 31 ORESTES sequences clustered with transcripts sequenced from that library.

### 3.4 Functional classification based on gene ontology assignments

**Fig. 2:**
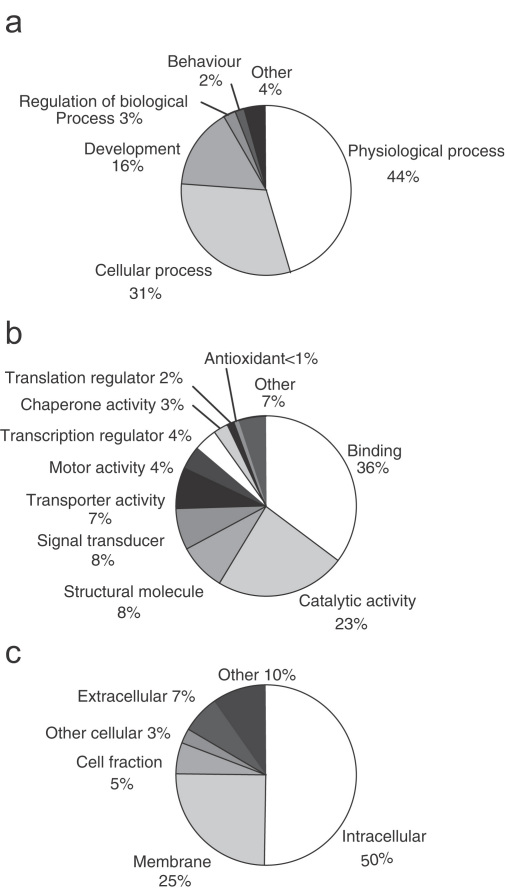
Gene ontologies. Percentage representation of gene ontology (GO) mappings for *B. glabrata* ESTs. (a) Biological processes, (b) molecular function and (c) cellular component. Note that individual GO categories can have multiple mappings and that the charts do not include ESTs with no blast/gene ontology matches.

The functions of the non-redundant 1843 sequences were assessed using gene ontology, based on Blast matches with genes whose functions have been previously assessed (Fig. 2). Of the 1843 ESTs, 587 were assigned a function, in three categories, biological process, molecular function and cellular component. In the biological process categories, the largest proportion (44%) was assigned to physiological processes (Fig. 2a). The most prevalent molecular functions (Fig. 2b) were binding (36%) and catalytic activity (23%). Other molecular function assignments were signal transducers (8%) and transcription regulators (4%), and five antioxidant genes (0.4%) were also identified. Over 80% of the cellular component assignments were for genes coding for cellular proteins including 50% intracellular and 25% membrane proteins (Fig. 2c). Gene ontologies were examined to identify genes that were homologous to antioxidant molecules, signalling molecules, transcriptional regulators, immune response genes and stress response genes (Table 3), since many of these might be significant in the snail's response to parasite infection. A total of 117 homologues of genes that code for proteins involved in cell signalling or transcriptional regulation were identified; these genes were categorised as follows: signal transducers (54), cell–cell signalling (19), transcription regulator activity (28), signal transducers and transcription factor regulators (10) (Table 3). Although not the original purpose of generating these ESTs, gene ontologies were also assessed by tissue type, strain, parasite susceptibility and whether parasite exposed or unexposed, for both biological process and molecular function (see supplementary material).

### 3.5 Functional classification based on KEGG pathway analysis

As an alternative method of categorising ESTs by biochemical function, clones were assigned to biochemical pathways using the KEGG website. Four hundred and thirteen ESTs were assigned to metabolic, genetic information processing, environmental information processing and cellular pathways (Tables 4 and 5). Thirty-one enzymes (38 clones) from 7 out of a possible 11 signal transduction pathways were identified as well as 25 enzymes (31 clones) from 7 out of a possible 9 immune-related pathways.

## 4 Discussion

Use of the ORESTES approach generated 1843 ESTs from different tissues and strains of *B. glabrata*. Only 3.4% of these had been previously characterized in *B. glabrata* and cluster analysis with other *B. glabrata* ESTs identified less than 300 clusters of overlapping sequences. Over half of the sequences showed no matches to previously sequenced genes in the non-redundant section of GenBank. Functional analysis of those with sequence similarity to previously characterized genes, using gene ontologies and KEGG assignments identified a number of antioxidant, signalling and transcriptional regulatory genes, molecules that may potentially be involved in snail/parasite interactions, as well as several immune and stress response proteins.

### 4.1 Antioxidant proteins

Four different genes were identified that were similar to molecules that demonstrate antioxidant functions in other organisms. Peroxinectin (CV548486) is a cell adhesion protein with peroxidase activity, which has been identified in other invertebrates including the crayfish *Pacifastacus leniusculus* [25], the black tiger shrimp *Penaeus monodon* [26], *Drosophila melanogaster* [27] and the white shrimp *Litopenaeus vannamei* [28], and is a functional equivalent of the vertebrate myeloperoxidase [25,29]. Peroxidasin (CV548777) is a similar protein with peroxidase activity associated with developmental processes in both *Drosophila* [30] and *Xenopus tropicalis* [31]. Dual oxidase 1 (Duox1) (CK149203), possesses a peroxidase domain and is thus categorized with antioxidant function; interestingly though, these transmembrane proteins also have a superoxide-generating subunit homologous to glycoprotein p91^phox^ [32], a host defence molecule that generates reactive oxygen species (ROS) [33,34]. Another Duox1 sequence has been identified in *B. glabrata* [17] but our sequence (CK149203) seems to be a paralog of this gene as it shows no similarity at the nucleotide level with the other sequences (CK989379, CK990069) and did not identify these sequences in BlastN searches, despite all matching the same section of translated protein in tBlastX searches.

### 4.2 Signalling molecules and transcriptional regulators

Based on our knowledge of other, well-characterised, biological systems, some of the identified signalling molecules and transcriptional regulators play a part in pathways that are likely to be involved in the innate immune response of snails. In a few cases (as described below), functional studies have shown that such signalling pathways contribute to the regulation of molluscan defence reactions.

One clone (EG030744) matched the transcription factor nuclear factor-*κ*B1 (NF-*κ*B1), a p105 NF-*κ*B subunit that is proteolytically processed to yield NF-*κ*B p50 [35]. The NF-*κ*B/Rel transcription factors comprise a family of evolutionarily conserved and structurally related proteins identified in a variety of vertebrates and invertebrates including the beetle *Allomyrina dichotoma* [36], the sea squirt *Ciona intestinalis* [37] and the bivalve mollusc *Crassostrea gigas* [38]. Such transcription factors are central to the NF-*κ*B pathway, a key intracellular pathway that co-ordinates the induction of defence genes in both mammals and *Drosophila*, and plays a pivotal role in vertebrate and invertebrate innate immunity [39,40]. I*κ*B kinase (IKK) complex associated protein (IKAP) [41] was also identified (EG030742). This protein contains potential IKK association sites and was thus originally thought to play a role in NF-*κ*B signalling by scaffolding the IKK signalsome [41]. Although this now seems unlikely (as discussed in [42]) IKAP seems to associate with stress-activated protein kinase/c-jun NH_2_-terminal kinase (SAPK/JNK) and regulate its activity in mammals [42]. Activation of SAPK/JNK occurs via the transmission of extracellular stress signals, and aside from the role that this protein plays in processes such as development, apoptosis, and proliferation, it can regulate immune responses in *Drosophila* [43,44]. Interestingly, SAPK/JNK is activated by recombinant human TNF-*α* in defence cells (haemocytes) of the bivalve mollusc *Mytilus galloprovincialis* [45] and, in the present study, we identified a homologue (clones CV548175, CV548723, CV548166, CV548685) of *Drosophila* JNK interacting protein 1 [46], a scaffold protein that aggregates specific components to form a functional SAPK/JNK signalling module in mammals [47].

Homologues of invertebrate integrin *α*_3_ [48] (CK149506), focal adhesion kinase (FAK) [49] (CK656717) and mammalian protein tyrosine kinase Src [50] (CK149232) were also identified. Integrins are a family of heterodimeric, transmembrane adhesion receptors whose ligand specificities are determined by the specific *α* and *β* subunits; integrins are crucial to cell adhesion and organisation of the actin cytoskeleton and they serve as important receptors in immune cell responses, cell migration and tissue integrity. Expressed in all metazoans, integrins have been characterized in several invertebrates [51], with *β*_1_ integrin subunits reported from haemocytes of the molluscs *C. gigas* [52] and *B. glabrata* [53]. Integrins nucleate the formation of focal adhesions and focal complexes and these events rely on the co-ordinated actions of signalling proteins that include FAK and Src. In mammals, integrin clustering is known to lead to autophosphorylation of FAK at Tyr^397^, FAK then associates with the SH2 domain of Src, which in turn phosphorylates FAK at Tyr^925^ [54]. In some cell types this can result in downstream signalling to the extracellular signal-regulated kinase (ERK) pathway [55], a signalling module that has been shown to regulate phagocytosis and nitric oxide production in haemocytes from *L. stagnalis* [56,57]. A recent study has demonstrated that integrin engagement results in increased phosphorylation of a FAK-like protein in *L. stagnalis* haemocytes and that integrin blockade inhibits phagocytosis and spreading by these cells [58]. Since integrins are also known to regulate cell spreading by haemocytes of *B. glabrata* [59] it appears that integrin binding is crucial to the defence responses of snails, particularly those involving actin remodelling. Thus, signalling through the identified FAK/Src proteins is likely to regulate such defence reactions, as has been shown in insects [60].

Protein kinase C (PKC) is known to play a role in regulating innate defences in mammals; this has also been documented for snails in which PKC seems to regulate nitric oxide (NO) and hydrogen peroxide (H_2_O_2_) production, phagocytosis and cell spreading by haemocytes [56,57,61,62]. In this context, it is interesting that we have now identified homologues of two proteins, activated protein kinase C receptor (RACK) (CK149425, CK149451) from *Xenopus* [63] and 14-3-3 *γ* (CO870195) from humans [64], which are known to interact with PKC. RACK has previously been characterized in *B. glabrata* by other workers [65], however, the nucleotide sequence fragment we identified differs significantly from that previously published, with only three short stretches being identified in a BlastN search with an E value of 3e-5. BlastX searches did identify (amongst other RACK sequences) the amino acid sequence of the previously sequenced *B. glabrata* RACK, with 89% similarity. Our nucleotide sequence also identified 12 other *B. glabrata* ESTs with close homology, so it seems likely that we have identified a second gene for RACK. 14-3-3 *γ* appears to be phosphorylated by PKC and might facilitate signalling to the ERK pathway via Raf [64]. *Biomphalaria glabrata* RACK might serve to direct the translocation of PKC isoforms to specific cellular compartments as it does in higher organisms [66].

We also identified a homologue of the B (regulatory) subunit of serine threonine protein phosphatase 2A (PP2A) [67] (CV548346), a heterotrimeric holoenzyme that either positively or negatively regulates the activities of wide variety of cellular signal transduction pathways including those involving IKK and ERK discussed above (for reviews see [68,69]). Also of interest are genes that were found to be homologous to those coding for proteins involved in protein kinase A and cAMP signalling, namely: adenylyl cyclase [70] (CV548064), the enzyme that generates the second messenger cAMP; cAMP-specific 3′,5′-cyclic phosphodiesterase (CV548523), an enzyme involved in cyclic nucleotide metabolism [71]; and the type N4 regulatory subunit of PKA [72] (CV548191). These proteins likely play a role in mollusc defence since the catecholamine noradrenaline modulates the phagocytic activity of *C. gigas* haemocytes via a *β*-adrenergic receptor-cAMP signalling pathway [73]. Finally, genes were found which matched to those of the transmembrane glycoprotein macrophage mannose receptor [74] (CO870241, CV548367), the Ras-related GTPase protein Rab 21 [75] (CK149518, CK149287) and hemolectin [76] (CV548237, CV548539, CV548566). The macrophage mannose receptor is a phagocytic receptor that targets pathogens such as bacteria and yeast which express mannose-rich glycoproteins [74] and Rab 21 has been recently found to interact with two LIM domain proteins in the slime mould *Dictyostelium* to collectively regulate phagocytosis [77]. The identified hemolectin showed homology to *Drosophila* hemolectin which is a major clot constituent in these flies [76].

### 4.3 Immune and stress response genes

Examination of the gene ontologies revealed 8 immune response genes and 10 response-to-stress genes. Of particular interest are *α* 2-macroglobulin (*α*2 M) and Rho-guanine nucleotide-exchange factor 4 (Rho-GEF 4). The identified *α*2 M (CV548690) is similar to that from the horseshoe crab *Limulus polyphemus* [78], a proteinase inhibitor similar to mammalian *α*2 M with broad reactivity towards proteinases; a similar inhibitor with activity towards serine, cysteine and metalloproteinase has been purified from *B. glabrata* plasma [79]. Such inhibitors could be important to defence, since they may be expressed during the humoral immune response in order to inactivate proteinases produced by invading pathogens [80]. The Rho-GEF 4 homologue (CO870295) was similar to that sequenced in humans [81]. This GEF is operational towards the small GTPases Rho A and Rac 1 and is thought to play a role in cell migration and cell–cell adhesion [82]. Given the universal nature of these cellular processes it is likely that Rho GEF 4 has a similar role in snails and thus may be important in the snail defence response towards pathogens.

## 5 Conclusions

The genes described above represent a set of those identified that might play important roles in molluscan defence. To eliminate pathogens such as parasites, the molluscan immune system must be able to mount a co-ordinated response to the invading organism, with processes such as cell adhesion and the production of reactive oxygen and nitrogen intermediates being crucial to the outcome of infection. Despite a parasite-mediated interference theory being proposed 25 years ago [83], the precise mechanism(s) by which schistosomes evade the defence response of their snail intermediate hosts remain largely unknown. A recent hypothesis paper has explored the idea that parasites might blunt the defence response of susceptible snails by interfering with key signal transduction pathways in their defence cells [84]; such a strategy could serve to alter gene expression and functional defence responses.

This EST gene discovery project has provided a significant number of genes for the first version of a custom *B. glabrata* cDNA microarray. A detailed investigation of the transcriptome in response to trematode infection in this snail intermediate host, in order to identify and understand the role of specific genes involved in the snail internal defence system can therefore now be carried out. Thus, we anticipate that through application of the microarray, we will move closer to gaining a comprehensive understanding of snail–schistosome interactions and the complex nature of the biological interplay that exists between snail and schistosome parasite.

## Figures and Tables

**Table 1:** *Biomphalaria glabrata* ORESTES libraries. The number of non-redundant (NR) sequences was determined after cluster and Blast analyses to remove duplicate and ribosomal sequences from within each library

Strain^a^	Tissue^b^	Parasite exposure^c^	N^o^. sequences obtained	% redundancy	N^o^. NR sequences	% Unique (and non-ribo)	Library name
1742	HF	EX	93	36.6	55	59.1	BgORESTES infected NHM 1742 Head/foot
1742	HF	UN	88	52.3	35	39.8	BgORESTES uninfected NHM 1742 Head/foot
1742	OT	UN	89	46.1	36	40.4	BgORESTES uninfected NHM 1742 Ovotestis
3017	OT	EX	94	50	45	47.9	BgORESTES schistosome-exposed NHM 3017 Ovotestis
1742	OT	EX	90	47.8	51	56.7	BgORESTES infected NHM 1742 Ovotestis 1
1742	OT	EX	91	58.2	37	40.7	BgORESTES infected NHM 1742 Ovotestis 2
1742	HAEM	EX	91	53.8	24	25.3	BgORESTES infected NHM 1742 Haemocytes
1742	HO	UN	92	51.1	42	45.7	BgORESTES uninfected NHM 1742 Haemopoietic organ
1742	HO	EX	96	56.3	36	37.5	BgORESTES infected NHM 1742 Haemopoietic organ
1742	HAEM	UN	92	58.7	24	26.1	BgORESTES uninfected NHM 1742 Haemocytes
3017	HF	EX	94	30.9	58	61.7	BgORESTES schistosome-exposed NHM 3017 Head/foot
3017	OT	UN	94	40.4	53	56.4	BgORESTES unexposed NHM 3017 Ovotestis
3017	OT	EX	93	40.9	52	55.9	BgORESTES schistosome-exposed NHM 3017 Ovotestis2
3017	HAEM	UN	94	47.7	35	37.2	BgORESTES unexposed NHM 3017 Haemocytes
3017	HAEM	EX	87	57.5	20	23.0	BgORESTES schistosome-exposed NHM 3017 Haemocytes
3017	HO	UN	91	28.6	56	61.5	BgORESTES unexposed NHM 3017 Haemopoietic organ
3017	HO	EX	81	30.9	47	58.0	BgORESTES schistosome-exposed NHM 3017 Haemopoietic organ
3017	HF	UN	95	34.7	52	54.7	BgORESTES unexposed NHM 3017 Head/foot
3017	HF	EX	96	52.1	34	35.4	BgORESTES schistosome-exposed NHM 3017 Head/foot2
3017	HAEM	UN	93	29	42	45.2	BgORESTES unexposed NHM 3017 Haemocytes2
3017	HAEM	EX	90	70	9	10.0	BgORESTES schistosome-exposed NHM 3017 Haemocytes2
3017	HO	UN	93	62.4	16	17.2	BgORESTES unexposed NHM 3017 Haemopoietic organ2
3017	HO	EX	96	71.9	20	20.8	BgORESTES schistosome-exposed NHM 3017 Haemopoietic organ2
3017	OT	UN	96	35.4	57	59.4	BgORESTES unexposed NHM 3017 Ovotestis2
3017	OT	EX	94	45.7	47	50.0	BgORESTES schistosome-exposed NHM 3017 Ovotestis3
3017	HF	UN	94	43.6	40	42.6	BgORESTES unexposed NHM 3017 Head/foot2
3017	HF	EX	93	50.5	39	41.9	BgORESTES schistosome-exposed NHM 3017 Head/foot3
1742	B	UN	95	20	66	69.5	BgORESTES uninfected NHM 1742 Brain
1742	B	EX	94	9.6	82	87.2	BgORESTES infected NHM 1742 Brain
3017	B	UN	91	71.4	18	19.8	BgORESTES unexposed NHM 3017 Brain
3017	B	EX	94	57.4	32	34.0	BgORESTES schistosome-unexposed NHM 3017 Brain
3032	HF	EX	95	51.6	43	45.3	BgORESTES infected NHM 3032 Head/foot
3032	OT	EX	94	46.8	47	50.0	BgORESTES infected NHM 3032 Ovotestis
3032	B	EX	94	42.6	49	52.1	BgORESTES infected NHM 3032 Brain
3032	HO	EX	96	36.5	56	58.3	BgORESTES infected NHM 3032 Haemopoietic organ
3032	HAEM	UN	95	24.2	64	67.4	BgORESTES uninfected NHM 3032 Haemocytes
3032	HAEM	EX	96	33.3	47	49.0	BgORESTES infected NHM 3032 Haemocytes
3032	HF	UN	93	36.6	59	63.4	BgORESTES uninfected NHM 3032 Head/foot
3032	OT	UN	94	19.1	74	78.7	BgORESTES uninfected NHM 3032 Ovotestis
3032	B	UN	95	23.2	73	76.8	BgORESTES uninfected NHM 3032 Brain
3032	HO	UN	93	16.1	71	76.3	BgORESTES uninfected NHM 3032 Haemopoietic organ
Total			3809		1843		
Mean				43.2	44.9	48.2	

a NHM Strain: 3017—resistant snails, 1742, 3032 (BB02)—susceptible snails.

b Tissue type: HF—head/foot, OT—ovotestis, HO—haemopoietic organ, HAEM—haemocytes, B—brain.

c Snails exposed (EX) or unexposed (UN) to *S. mansoni* miracidia.

**Table 2:** Blast results summary. Breakdown of the types of sequences obtained from the *B. glabrata* ORESTES libraries identified with Blast searches of GenBank

	All sequences		Non-redundant sequences	
Category	N^o^. sequences	% sequences	N^o^. sequences	% sequences
Protein	907	23.8	739	40.1
Mitochondrial Protein	4	0.1	4	0.2
Ribosomal Protein	14	0.4	11	0.6
*Biomphalaria* Protein	35	0.9	22	1.2
*Biomphalaria* fragment	41	1.1	40	2.2
Ribosomal	1482	38.9	44	2.4
Unknown (no BLAST match)	1106	29.0	832	45.1
Unknown EST	84	2.2	56	3.0
Unknown Protein	127	3.3	88	4.8
Other	8	0.2	7	0.4
Total	3809	100.0	1843	100.0

**Table 3:** Transcripts selected by gene ontology. Individual *B. glabrata* ESTs that identified antioxidant proteins, signal transducers, transcription regulators and immune or stress response proteins

Name	GenBank accession	Gene ID (Blast result)^a^	Organism^a^	Blast score^a^
*Antioxidant* (GO:0016209)				
3032HOUN59B8	CV548777	Peroxidasin (mKIAA0230) [BAC65505]	*M. musculus*	2E-23
1742HFEX1H9	CK149203	Dual oxidase 1 [Q8HZK3]	*S. scrofa*	2E-31
3017OTEX4H6	CK149399	Putative iron dependent peroxidase [Q8XGB1]	*S. typhi*	5E-15
1742OTEX5B12	CK149417	Putative iron dependent peroxidase [Q8XGB1]	*S. typhi*	7E-11
3032HAEMEX55E1	CV548486	Peroxinectin [AAL05973]	*P. monodon*	6E-17
*Signal transducers* (GO: 0007165)				
3032BEX52G8	CV548350	Filamin 1 [P21333]	*H. sapiens*	5E-53
1742BUN46B6	CV548064	Adenylyl cyclase [Q9QW33]	*Rattus sp.*	4E-78
3017OTUN34C11	CO870317	Ankyrin 2 (Brain ankyrin) [Q01484]	*H. sapiens*	2E-11
3017HFEX11B10	CK149239	Inhibitor of apoptosis protein [Q8UWD2]	*D. rerio*	4E-17
3032OTUN57G6	CV548625	Inhibitor of apoptosis protein [Q8UWD2]	*D. rerio*	4E-11
3017BEX49B1	CV548199	Buccalin precursor [P20481]	*A. californica*	5E-67
3032BEX52A2	CV548323	Buccalin precursor [P20481]	*A. californica*	2E-54
3032BEX52A12	CV548364	Buccalin precursor [P20481]	*A. californica*	2E-19
3032BUN58E5	CV548692	Buccalin precursor [P20481]	*A. californica*	5E-46
3032BUN58B9	CV548713	Buccalin precursor [P20481]	*A. californica*	2E-19
3032HAEMEX55A11	CV548523	cAMP-specific 3’,5’-cyclic phosphodiesterase [P12252]	*D. melanogaster*	2E-31
3032OTUN57A5	CV548614	Serine/threonine-protein kinase TNNI3 K (ANK repeats) [Q7TQP6]	*R. norvegicus*	1E-18
3032OTUN57B1	CV548590	Regulator of G-protein signalling 22 [Q9BYZ4]	*H. sapiens*	9E-22
3032HOUN59G3	CV548755	Bent (GH07636p) [Q9V4F7]	*D. melanogaster*	2E-19
3032HOUN59B1	CV548737	Bent (GH07636p) [Q9V4F7]	*D. melanogaster*	3E-76
3032HOEX53H6	CV548394	Elongation factor 1 alpha [P13549]	*X. laevis*	1E-48
3017BUN48A11	CV548195	FMRFamide neuropeptides [P19802]	*L. stagnalis*	4E-45
3032BUN58F5	CV548693	FMRFamide neuropeptides [P19802]	*L. stagnalis*	1E-34
3017OTEX17H11	CK656694	Myotilin (Titin immunoglobulin domain protein) [Q9UBF9]	*H. sapiens*	8E-13
3017HAEMUN28C9	CO870195	Protein kinase C inhibitor 1 (14-3-3-like) [P35214]	*H. sapiens*	3E-52
3032HFEX50F2	CV548237	Hemolectin [Q9U5D0]	*D. melanogaster*	2E-20
3032HFUN56E2	CV548539	Hemolectin [Q9U5D0]	*D. melanogaster*	2E-20
3032HFUN56B8	CV548566	Hemolectin [Q9U5D0]	*D. melanogaster*	4E-28
3017BEX49E3	CV548205	Molluscan insulin-related peptide 3 [P80090]	*L. stagnalis*	6E-13
3032HFUN56E6	CV548560	Multiple EGF-like-domain protein 3 [O75095]	*H. sapiens*	1E-30
3032HFUN56F9	CV548575	Multiple EGF-like-domain protein 3 [O75095]	*H. sapiens*	8E-24
3032HOUN59C2	CV548745	Nidogen 1 [P10493]	*M. musculus*	2E-23
3017HAEMEX19E1	CK656743	Feline leukemia virus subgroup C receptor [Q9N1F2]	*F. catus*	2E-28
3017HOUN20E11	CK656783	PERQ amino acid rich, with GYF domain 1 [Q99MR1]	*M. musculus*	1E-23
3032HFEX50E11	CV548269	PERQ amino acid rich, with GYF domain 1 [Q99MR1]	*M. musculus*	1E-20
3017HFUN44E6	CO870392	Polycystic kidney disease protein 2 [Q7Z2B5]	*S. purpuratus*	9E-45
3032HOUN59B11	CV548796	Polydom protein precusor [Q9ES77]	*M. musculus*	3E-12
3017HAEMUN28F10	CO870213	Polyserase 1B protein [Q7Z410]	*H. sapiens*	3E-22
3017HAEMEX29B4	CO870226	Polyserase 1B protein [Q7Z410]	*H. sapiens*	1E-22
3032BEX52E7	CV548346	Serine/threonine protein phosphatase 2A [P11493]	*S. scrofa*	4E-63
3032OTUN57F6	CV548624	pRb-interacting protein RbBP-36 [Q8IZZ0]	*H. sapiens*	1E-20
3032BEX52H9	CV548355	Tyrosine phosphatase IA-2beta [Q9Y4I9]	*H. sapiens*	6E-57
3032BUN58D5	CV548691	Tyrosine phosphatase IA-2beta [Q9Y4I9]	*H. sapiens*	2E-46
3032OTUN57B12	CV548658	Muscle M-line assembly protein UNC-89 [O01761]	*C. elegans*	2E-12
3032HOUN59F4	CV548760	Muscle M-line assembly protein UNC-89 [O01761]	*C. elegans*	8E-25
3032HOEX53B12	CV548417	Muscle M-line assembly protein UNC-89 [O01761]	*C. elegans*	2E-20
1742HOUN8B5	CK149518	RAS related protein Rab21 [Q9UL25]	*H. sapiens*	3E-29
3017HFEX11H12	CK149287	RAS related protein Rab21 [Q9UL25]	*H. sapiens*	1E-29
1742HFEX1G2	CK149192	GTP-binding nuclear protein Ran [P79735]	*D. rerio*	2E-80
3017OTUN34B9	CO870310	Serine/threonine protein kinase SSTK [Q9BXA6]	*H. sapiens*	3E-18
3032BUN58F11	CV548727	E3 ubiquitin-protein ligase HECTD1 [Q9ULT8]	*H. sapiens*	6E-66
3032HOEX53G8	CV548401	SNF4/AMP-activated protein kinase gamma subunit [O96613]	*D. melanogaster*	2E-29
3017OTEX17B2	CK656650	Transportin-SR [Q9Y540]	*H. sapiens*	9E-58
1742HFEX1H2	CK149197	Twitchin [Q7YT99]	*M. galloprovincialis*	5E-28
3017BUN48G6	CV548191	Type N4 regulatory subunit of protein kinase A [P31319]	*A. californica*	1E-50
3032OTUN57C10	CV548648	Testis-enriched protein tyrosine phosphatase [Q9WU22]	*M. musculus*	2E-66
1742HAEMEX7G1	CK149506	Integrin alpha 3 [Q86G86]	*P. includens*	8E-23
3032OTUN57G7	CV548631	Receptor type protein-tyrosine phosphatase T precursor [O14522]	*H. sapiens*	3E-16
3017HAEMEX19A3	CK656730	G protein-coupled receptor kinase type 2 [Q9U756]	*H. americanus*	3E-62
3017HFEX45B5	CO870422	Megalin [P98164]	*H. sapiens*	6E-20
1742OTEX5D3	CK149425	Activated protein kinase c receptor [Q9W7I1]	*X. laevis*	1E-114
1742OTEX5H8	CK149451	Activated protein kinase c receptor [Q9W7I1]	*X. laevis*	8E-24
3032HOUN59A5	CV548763	Receptor type guanylyl cyclase [Q9BPR0]	*B. mori*	1E-22
3032BUN58B3	CV548678	Soluble guanylyl cyclase alpha [Q7YW37]	*L. marginatus*	4E-63
1742HOEX9C10	CK149566	Collagen alpha 1(XIV) chain precusor (Undulin) [P32018]	*G. gallus*	7E-15
1742BEX47A12	CV548175	JNK interacting protein 1 [Q9W0K0]	*D. melanogaster*	4E-53
3032BUN58B11	CV548723	JNK interacting protein 1 [Q9W0K0]	*D. melanogaster*	5E-31
1742BEX47E10	CV548166	JNK interacting protein 1 [Q9W0K0]	*D. melanogaster*	2E-27
3032BUN58E4	CV548685	JNK interacting protein 1 [Q9W0K0]	*D. melanogaster*	8E-24
3032BUN58A3	CV548677	Serine/threonine kinase receptor type1 [O73801]	*T. rubripes*	1E-57
3032HFEX50H7	CV548257	Plectin [Q15149]	*H. sapiens*	8E-15
3032HOEX53F1	CV548369	Smad anchor for receptor activation (SARA) [Q9YHB9]	*X. laevis*	1E-56
3017HFEX11A5	CK149232	Src-family protein tyrosine kinase [Q8WQM5]	*S. purpuratus*	4E-51
3032HAEMEX55D11	CV548524	TNF receptor-associated factor 1 [Q13077]	*H. sapiens*	2E-27
3017HFEX45B8	CO870431	Epidermal growth factor precusor [P01133]	*H. sapiens*	7E-22
3017HFEX45A4	CO870419	Fibrillin [P35555]	*H. sapiens*	8E-26
3017HOUN32A5	CO870241	Macrophage mannose receptor [Q61830]	*M. musculus*	2E-14
3032HOEX53A1	CV548367	Macrophage mannose receptor [Q61830]	*M. musculus*	9E-23
1742HOUN8F1	CK149540	CYR61 protein precursor [Q9ES72]	*R. norvegicus*	4E-29
3017HAEMUN18E5	CK656717	Focal adhesion kinase [Q7Z1D3]	*L. variegatus*	1E-45
*Cell-cell signalling* (GO: 0007267)				
3032HAEMUN54B12	CV548481	Nicotinic acetylcholine receptor Dalpha6 [Q8T7S2]	*D. melanogaster*	1E-49
1742HOEX9F4	CK149578	Afadin (AF-6 protein) [P55196]	*H. sapiens*	2E-18
3017HAEMUN18H12	CK656729	Glutamate Receptor 2 [Q10914]	*C. elegans*	1E-15
3017HOUN20E9	CK656782	Bone morphogenetic protein 10 preproprotein [Q9R229]	*M. musculus*	3E-30
3017HOEX21A5	CK656808	Bone morphogenetic protein 10 preproprotein [Q9R229]	*M. musculus*	3E-30
3032HFUN56E10	CV548580	Clathrin heavy chain [P11442]	*H. sapiens*	1E-119
3032BEX52G5	CV548337	GABA Transaminase [P50554]	*R. norvegicus*	1E-28
3032BUN58G12	CV548734	Cadherin-related tumour suppressor [Q14517]	*H. sapiens*	4E-28
3017HFEX45B11	CO870445	Guanylate kinase associated protein [O14490]	*H. sapiens*	4E-22
1742BUN46F12	CV548100	Guanylate kinase associated protein [O14490]	*H. sapiens*	4E-21
3032HOUN59G12	CV548804	Synaptojanin 2 [O15056]	*H. sapiens*	5E-13
1742BUN46H8	CV548080	Kinesin-like protein KIF1A [Q12756]	*H. sapiens*	3E-30
1742BUN46F1	CV548040	Kinesin-like protein KIF1B [Q8R524]	*R. norvegicus*	1E-108
1742HFEX1H4	CK149199	Lethal giant larvae homolog 1 [O00188]	*H. sapiens*	7E-43
1742BUN46E9	CV548085	Prohormone convertase 2 (LPC2) [Q25409]	*L. stagnalis*	1E-128
3032BUN58A9	CV548712	Munc13-2 protein [Q62769]	*R. norvegicus*	1E-16
3017HFEX45B3	CO870416	Munc13-2 protein [Q62769]	*R. norvegicus*	3E-39
1742HOEX9G9	CK149583	Sodium/potassium-transporting ATPase alpha-1 chain [Q9DGL6]	*D. rerio*	6E-81
3017BEX49D12	CV548229	Synaptotagmin 11 [O08835]	*R. norvegicus*	9E-50
3017BUN48C2	CV548182	Synaptotagmin 11 [O08835]	*R. norvegicus*	3E-33
3032BUN58D7	CV548701	Synaptotagmin 11 [O08835]	*R. norvegicus*	4E-34
3032OTUN57B8	CV548634	Tyrosine-protein kinase receptor [Q06807]	*B. taurus*	6E-61
3032BUN58C12	CV548731	Putative pyrokinin receptor [Q7RTK4]	*A. gambiae*	5E-26
1742BEX47H10	CV548168	Tryptophan hydroxylase [Q9NJQ3]	*L. stagnalis*	6E-41
3032BUN58D3	CV548680	Tryptophan hydroxylase [Q9NJQ3]	*L. stagnalis*	1E-40
*Transcription regulator activity* (GO: 0030528)				
3017BEX49A4	CV548206	Eukaryotic translation intiation factor 3 subunit 10 [Q8I5S6]	*P. falciparum*	2E-21
3017OTEX17B6	CK656654	Calreticulin [Q26268]	*A. californica*	2E-63
1742HFEX1G4	CK149194	Chromodomain helicase DNA binding protein 5 [Q8TDI0]	*H. sapiens*	7E-55
3017OTEX17C2	CK656662	C-terminal binding protein [O46036]	*D. melanogaster*	1E-79
1742HOUN8D7	CK149529	Cytochrome P450 monooxygenase [O04892]	*N. tabacum*	7E-21
1742HAEMUN10F7	CK149220	Cytochrome P450 monooxygenase [O04892]	*N. tabacum*	4E-17
1742HOUN8F12	CK149546	Cytochrome P450 monooxygenase [O04892]	*N. tabacum*	3E-15
3017HFEX45H10	CO870443	Elongation factor-2 [P13639]	*H. sapiens*	3E-49
3017OTUN16F11	CK656633	Embryonic ectoderm development protein [P97462]	*M. musculus*	2E-51
3032OTUN57E7	CV548630	HemK methyltransferase family member [Q9Y5R4]	*H. sapiens*	3E-30
3017OTUN34G10	CO870315	High mobility group protein [P40618]	*G. gallus*	2E-11
1742OTEX5D12	CK149429	Homeodomain interacting protein kinase 2 [Q9H2×6]	*H. sapiens*	1E-57
3017OTEX17A8	CK656647	Fragile-chorion membrane protein [P13709]	*D. melanogaster*	6E-27
1742BUN46A9	CV548081	LIM domain protein BX (BEADEX protein). [P91608]	*Drosophila* sp.	2E-60
1742BEX47F11	CV548173	LIM domain protein BX (BEADEX protein). [P91608]	*Drosophila* sp.	1E-60
3017HFEX45E11	CO870447	Max dimerization protein 1; mad [Q05195]	*H. sapiens*	7E-24
3032OTUN57C3	CV548602	Myeloid/lymphoid or mixed-lineage leukemia protein 2 [O14686]	*H. sapiens*	4E-59
3017HAEMUN28B11	CO870190	Bifunctional aminoacyl-tRNA synthetase [P28668]	*D. melanogaster*	2E-56
3017OTUN16C3	EG030744	Nuclear factor NF-kB1 [P25799]	*M. musculus*	1E-17
3017HFUN44C3	CO870383	LReO_3 protein [Q8UUM8]	*O. latipes*	4E-16
3032HOEX53E9	CV548405	LReO_3 protein [Q8UUM8]	*O. latipes*	1E-18
3032HFEX50B11	CV548268	LReO_3 protein [Q8UUM8]	*O. latipes*	2E-18
1742HOUN8F2	CK149541	LReO_3 protein [Q8UUM8]	*O. latipes*	2E-31
3017OTEX35D8	CO870357	Transcriptional activator protein Pur-alpha [P42669]	*M. musculus*	7E-31
1742HAEMUN10D9	CK149214	Retinoblastoma binding protein 5 [Q15291]	*H. sapiens*	1E-61
3032HFEX50F6	CV548253	Smad4 type2 [Q9W639]	*X. laevis*	7E-76
1742HAEMEX7F10	CK149504	Transcription elongation factor DSIF [O00267]	*H. sapiens*	1E-94
3017HOUN32D1	CO870235	Transcription elongation factor DSIF [O00267]	*H. sapiens*	8E-32
3032HOEX53F10	CV548409	RUSH-1 [Q95216]	*O. cuniculus*	9E-68
1742HFEX1H6	CK149201	Tis11 family protein [P47974]	*H. sapiens*	3E-36
3017HOEX33B8	CO870263	Transcription factor IID p80 chain homolog [Q91857]	*X. laevis*	2E-55
1742HFEX1C3	CK149163	Tropomyosin [O97192]	*H. aspersa*	6E-39
3017HOEX21A2	CK656807	Tropomyosin [O97192]	*H. aspersa*	1E-40
3017HFUN44E7	CO870396	Tropomyosin [O97192]	*H. aspersa*	7E-77
3032HAEMUN54H4	CV548439	Staphylococcal nuclease domain-containing protein 1 [Q7K2F4]	*H. sapiens*	3E-26
3032HAEMUN54E4	CV548436	Winged-helix repressor FOXP4 [Q8CIS1]	*M. musculus*	3E-12
3032HAEMUN54C3	CV548430	Jumonji domain containing protein 2C [Q9H3R0]	*H. sapiens*	5E-73
*Signal transducers and transcription factor regulators* (both GO: 0007165 and GO: 0030528)				
1742OTEX5C1	CK149418	WDR9protein [Q9NSI6]	*H. sapiens*	1E-80
3017HFEX11G11	CK149281	Pliotropic regulator 1 [Q9WUC8]	*R. norvegicus*	2E-29
1742BUN46G3	CV548048	Transcriptional regulator ATRX protein [P46100]	*H. sapiens*	7E-90
1742BEX47D9	CV548159	Beta-catenin [P35224]	*U. caupo*	2E-34
3032HOEX53H9	CV548408	Beta-catenin [P35224]	*U. caupo*	2E-83
1742BUN46H7	CV548074	Fibropellin-1 [P10079]	*S. purpuratus*	1E-17
3032OTUN57B7	CV548627	HIRA protein [P54198]	*H. sapiens*	3E-68
1742OTEX5A12	EG030742	IKAP [O95163]	*H. sapiens*	4E-41
1742HFUN2A2	CK149289	Nuclear hormone receptor FTZ-F1 beta [Q05192]	*D. melanogaster*	2E-17
3032HOUN59B7	CV548771	Orphan nuclear receptor NR1D2 [Q14995]	*H. sapiens*	4E-25
1742OTEX5C2	CK149419	Histone-binding protein RBBP4 [Q09028]	*H. sapiens*	1E-131
*Immune response* (GO: 0006955)				
3032BUN58C5	CV548690	Alpha 2-macroglobulin [O01717]	*Limulus sp.*	5E-20
1742OTEX6D7	CK149467	Alpha-1 inhibitor III [Q62591]	*R. sordidus*	3E-16
1742BEX47H4	CV548126	ATP-binding cassette sub-family F member 1 [Q8NE71]	*H. sapiens*	5E-56
1742HOUN8H9	CK149553	Paramyosin [O96064]	*M. galloprovincialis*	7E-61
3017OTUN16E12	CK656627	Exosome complex exonuclease RRP45 [Q9JHI7]	*M. musculus*	5E-73
3032BEX52B6	CV548339	Exosome complex exonuclease RRP45 [Q9JHI7]	*M. musculus*	2E-77
1742OTUN3F6	CK149346	J kappa-recombination signal binding protein [P31266]	*M. musculus*	2E-24
3017OTUN34C5	CO870295	Rho guanine nucleotide exchange factor 4 [Q9NR80]	*H. sapiens*	4E-36
1742BEX47G7	CV548147	Soma ferritin [P42577]	*L. stagnalis*	7E-60
3032HFUN56G3	CV548545	Soma ferritin [P42577]	*L. stagnalis*	3E-59
*Response to stress* (GO: 0006950)				
3017HOUN20F4	CK656786	60S acidic ribosomal protein P0 [Q9NHP0]	*S. crassipalpis*	9E-73
1742HOEX9H9	CK149589	Cdc7-related kinase [Q9Z2Y5]	*M. musculus*	4E-45
3032HAEMUN54A6	CV548444	ATP-dependent RNA helicase Ddx1 [Q9VNV3]	*D. melanogaster*	1E-60
3017OTUN34C6	CO870298	78 kDa glucose-regulated protein precursor [Q16956]	*A. californica*	2E-86
3017BUN48H5	CV548188	Helicase-like protein NHL [Q9NZ71]	*H. sapiens*	1E-25
3032OTUN57E6	CV548623	Hypoxia up-regulated 1 [Q9Y4L1]	*H. sapiens*	1E-38
3032HOEX53A9	CV548403	Mismatch repair protein pms1 homologue [Q8IBJ3]	*P. falciparum*	1E-14
3017HOEX33D3	CO870252	DNA mismatch repair protein Mlh1 [P40692]	*H. sapiens*	2E-25
1742OTEX6D1	CK149464	Polyubiquitin [P62988]	*H. sapiens*	2E-55
3017OTUN34G6	CO870301	Polyubiquitin [P62988]	*H. sapiens*	6E-85
1742HOUN8C5	CK149524	Polyubiquitin [P62988]	*H. sapiens*	8E-44

a GenBank accession number, organism and E value given for the top match.

**Table 4:** KEGG pathways identified by *B. glabrata* ESTs

	KEGG categories represented	Enzymes^a^	Clones^b^
1	*Metabolism*		
1.1	*Carbohydrate metabolism*		
1.1.1	Glycolysis/gluconeogenesis	7	12
1.1.3	Pentose phosphate pathway	4	5
1.1.5	Fructose and mannose metabolism	1	2
1.1.6	Galactose metabolism	2	2
1.1.7	Ascorbate and aldarate metabolism	3	4
1.1.8	Starch and sucrose metabolism	6	12
1.1.9	Aminosugars metabolism	1	1
1.1.11	Pyruvate metabolism	4	5
1.1.12	Glyoxylate and dicarboxylate metabolism	1	1
1.1.13	Propanoate metabolism	2	2
1.1.14	Butanoate metabolism	5	5
1.1.17	Inositol phosphate metabolism	1	1
1.2	*Energy metabolism*		
1.2.1	Oxidative phosphorylation	7	7
1.2.2	ATP synthesis	1	1
1.2.4	Carbon fixation	3	7
1.2.6	Methane metabolism	2	2
1.2.7	Nitrogen metabolism	1	1
1.3	*Lipid metabolism*		
1.3.1	Fatty acid biosynthesis	1	1
1.3.2	Fatty acid elongation in mitochondria	1	2
1.3.3	Fatty acid metabolism	2	2
1.3.4	Synthesis and degradation of ketone bodies	1	1
1.3.6	Bile acid biosynthesis	1	1
1.3.9	Glycerolipid metabolism	1	1
1.3.10	Glycerophospholipid metabolism	2	2
1.4	*Nucleotide metabolism*		
1.4.1	Purine metabolism	6	8
1.4.2	Pyrimidine metabolism	3	4
1.5	*Amino acid metabolism*		
1.5.1	Glutamate metabolism	2	2
1.5.2	Alanine and aspartate metabolism	1	1
1.5.3	Glycine, serine and threonine metabolism	4	6
1.5.4	Methionine metabolism	1	2
1.5.6	Valine, leucine and isoleucine degradation	2	2
1.5.7	Valine, leucine and isoleucine biosynthesis	2	3
1.5.8	Lysine biosynthesis	1	1
1.5.9	Lysine degradation	2	2
1.5.10	Arginine and proline metabolism	5	7
1.5.11	Histidine metabolism	2	3
1.5.12	Tyrosine metabolism	2	3
1.5.13	Phenylalanine metabolism	2	2
1.5.14	Tryptophan metabolism	5	6
1.5.15	Phenylalanine, tyrosine and tryptophan biosynthesis	2	3
1.5.16	Urea cycle and metabolism of amino groups	3	4
1.6	*Metabolism of other amino acids*		
1.6.1	*β*-alanine metabolism	3	4
1.6.3	Aminophosphonate metabolism	1	2
1.6.4	Selenoamino acid metabolism	1	2
1.7	*Glycan biosynthesis and metabolism*		
1.7.1	*N*-Glycan biosynthesis	1	1
1.7.3	*N*-Glycan degradation	1	1
1.7.4	*O*-Glycan biosynthesis	1	1
1.7.12	Glycosphingolipid metabolism	1	1
1.8	*Biosynthesis of polyketides and nonribosomal peptides*		
1.8.3	Biosynthesis of ansamycins	1	1
1.9	*Metabolism of cofactors and vitamins*		
1.9.3	Vitamin B6 metabolism	2	2
1.9.6	Biotin metabolism	1	1
1.9.7	Folate biosynthesis	3	8
1.9.10	Porphyrin and chlorophyll metabolism	3	4
1.9.11	Ubiquinone biosynthesis	4	4
1.10	*Biosynthesis of secondary metabolites*		
1.10.4	Limonene and pinene degradation	2	2
1.10.6	Stilbene, coumarine and lignin biosynthesis	3	4
1.10.8	Alkaloid biosynthesis I	1	2
1.10.12	Streptomycin biosynthesis	1	1
1.11	*Biodegradation of xenobiotics*		
1.11.4	*γ*-hexachlorocyclohexane degradation	2	2
1.11.5	3-chloroacrylic acid degradation	1	2
1.11.6	1,1,1-trichloro-2,2-bis(4-chlorophenyl)ethane (DDT) degradation	1	2
1.11.8	1,2-dichloroethane degradation	1	1
1.11.14	Fluorene degradation	2	3
1.11.17	Benzoate degradation via hydroxylation	1	2
1.11.18	Atrazine degradation	1	1
1.11.20	1- and 2-methylnaphthalene degradation	1	2
2	*Genetic information processing*		
2.1	*Transcription*		
2.1.2	RNA polymerase	1	1
2.1.3	Basal transcription factors	2	2
2.2	*Translation*		
2.2.2	Ribosome	20	27
2.2.3	Aminoacyl-tRNA biosynthesis	16	35
2.3	*Folding, sorting and degradation*		
2.3.1	Protein export	2	3
2.3.2	Type II secretion system	1	1
2.3.7	Ubiquitin mediated proteolysis	3	4
2.3.8	Proteasome	7	11
3	*Environmental information processing*		
3.1	*Membrane transport*		
3.1.1	ABC transporters	15	23
3.2	*Signal transduction*		
3.2.1	Two-component system	3	3
3.2.2	MAPK signalling pathway	4	7
3.2.3	Wnt signalling pathway	6	6
3.2.4	Notch signalling pathway	1	1
3.2.6	TGF-*β* signalling pathway	5	5
3.2.9	Calcium signalling pathway	10	14
3.2.10	Phosphatidylinositol signalling system	2	2
3.3	*Signalling molecules and interaction*		
3.3.1	Neuroactive ligand–receptor interaction	17	20
3.3.2	Cytokine–cytokine receptor interaction	3	3
3.3.3	ECM–receptor interaction	5	7
3.3.4	Cell adhesion molecules (CAMs)	25	36
4	*Cellular processes*		
4.1	*Cell motility*		
4.1.3	Regulation of actin cytoskeleton	15	37
4.2	*Cell growth and death*		
4.2.1	Cell cycle	5	5
4.2.2	Apoptosis	6	11
4.3	*Cell communication*		
4.3.1	Focal adhesion	13	26
4.3.2	Adherens junction	8	16
4.3.3	Tight junction	9	21
4.3.4	Gap junction	6	25
4.4	*Endocrine system*		
4.4.1	Insulin signalling pathway	6	8
4.4.2	Adipocytokine signalling pathway	3	3
4.5	*Immune system*		
4.5.1	Hematopoietic cell lineage	2	2
4.5.2	Complement and coagulation cascades	5	7
4.5.3	Toll-like receptor signalling pathway	1	1
4.5.4	Natural killer cell mediated cytotoxicity	2	2
4.5.6	T cell receptor signalling pathway	2	2
4.5.7	B cell receptor signalling pathway	6	7
4.5.9	Leukocyte transendothelial migration	7	10
4.8	*Development*		
4.8.2	Axon guidance	7	10
4.9	*Behaviour*		
4.9.1	Circadian rhythm	3	7

a Enzymes—the number of enzymes from each pathway that were identified.

b Clones—the number of ORESTES clones that identified enzymes.

**Table 5:** Summary of KEGG pathways identified by *B. glabrata* ESTs

		Pathways represented	Total possible	Enzymes identified	No. EST clones
*Metabolic pathway*					
1.1	Carbohydrate metabolism	13	17	37	52
1.2	Energy metabolism	5	8	14	18
1.3	Lipid metabolism	7	12	9	10
1.4	Nucleotide metabolism	2	2	9	12
1.5	Amino acid metabolism	15	16	36	47
1.6	Metabolism of other amino acids	3	9	5	8
1.7	Glycan biosynthesis and metabolism	4	19	4	4
1.8	Biosynthesis of polyketides and nonribosomal peptides	1	9	1	1
1.9	Metabolism of cofactors and vitamins	5	11	13	19
1.10	Biosynthesis of secondary metabolites	4	16	7	9
1.11	Biodegradation of xenobiotics	8	21	10	15
		67	140	145	195
*Genetic information processing*					
2.1	Transcription	2	3	3	3
2.2	Translation	2	3	36	62
2.3	Folding, sorting and degradation	4	8	13	19
2.4	Replication and repair	0	1	0	0
		8	15	52	84
*Environmental information processing*					
3.1	Membrane transport	1	2	15	23
3.2	Signal transduction	7	11	31	38
3.3	Signalling molecules and interaction	4	4	50	66
		12	17	96	127
*Cellular processes*					
4.1	Cell motility	1	3	15	37
4.2	Cell growth and death	2	2	11	16
4.3	Cell communication	4	4	36	88
4.4	Endocrine system	2	3	9	11
4.5	Immune system	7	9	25	31
4.6	Nervous system	0	2	0	0
4.7	Sensory system	0	2	0	0
4.8	Development	1	2	7	10
4.9	Behaviour	1	1	3	7
		18	28	106	200
