# Supplementary material for: Biomphalaria glabrata transcriptome: Identification of cell-signalling, transcriptional control and immune-related genes from open reading frame expressed sequence tags (ORESTES)
Source: Dev Comp Immunol. 2007;31(8):763–82. doi: 10.1016/j.dci.2006.11.004 (PMC1871615; doi:10.1016/j.dci.2006.11.004)
Supplement: Supplementary file 1 — Online Supplementary Materials [file mmc1.doc]

| Name | Sequence |
| --- | --- |
| ESTA | CTATGGTTATAGGGATAC |
| ESTB | CTAAGTATGTCCTGTTCC |
| ESTC | TTTCAACTAATAGCAATC |
| ESTD | CTACCACTAACTATTCTC |
| ESTE | GGTCTCGTTCAACTCTTC |
| ESTF | CACAGTCAACCCACTGAG |
| ESTG | CAAGGTTGGTTTACTGAG |
| ESTH | CTCTTTACTGGCAAACAC |
| ESTI | GGCTATAGTCAAGAGTGG |
| ESTJ | GTCAGAAAGCCTTTGAAG |
| ESTK | CCCTAAAAAGAGCACCTC |
| ESTL | GCTCATGCATTAGTGATG |
| ESTM | GGTTCGTATAGTAGAGTTG |
| ESTN | CGGAAAGACCCTAAGAGC |
| ESTO | GTCTGTCCCATGTACACC |
| ESTP | CAAAGAGCCAACCTAAACG |
| ESTQ | GCTATGTAAGGACACAAC |
| ESTR | CATCCGGATGCTATAGGC |
| ESTS | GGTTAGGTTTATTTAGAG |
| ESTT | CCACTACAACTGTATAAC |
| ESTB2 | TACGCCACTATGAAACATTG |
| ESTD2 | AGAATGGACACATCAAAGG |
| ESTF2 | CTGCCCCGTACGCTAC |
| ESTH2 | CAATGAGCCCGACAGC |
| ESTA2 | ATTATGTTGTTTGATGGAGTTA |
| ESTU | CTACTTTGGATACGATAC |
| ESTV | CTTTTCCACCATCTATAC |
| ESTX | ACTTAGCCTACCTGTAAG |
| ESTY | GATTACAGGTCACCAATG |
| ESTZ | GTTTGAGGGCATCACATG |
| ESTAA | GACAACAGTGTATGTTGG |
| ESTAB | CACTGTTGTGTGGTTTAC |
| ESTAC | CTCTATCTGATAAACGAC |
| ESTAD | CCTGCTGCAACTAGTGTG |
| ESTAE | TCACAAACCAAGTGTCATG |
| ESTAF | CACCCGAATAGAACCTGTG |
| ESTAG | CCATTAAGGGATTGTTAGC |
| ESTAH | CCACTACTCTTTCAAATGG |
| ESTAI | CCATGTTACGACTTATCTC |
| ESTAJ | GCAGCCAATTAATCCTAAC |
| EST1 | ACTGTGTCTAGTGTGAGG |
| EST2 | TTCTTCGAGGCAACACAG |
| EST3 | CGTGAATGAATGGAGAGG |
| EST4 | CCATAGTGTGCGAAAGAG |
| EST5 | TGCACATGGTTAGTCTGG |
| EST6 | GGAGCAATACGCATCAAG |
| EST7 | CGTTCGGCGTGGACATAG |
| EST8 | TACCCTCCGGCTGGTTAG |
| EST9 | GTAGCGCTAGTGGTAGGG |
| EST10 | GGCGGGACTGATAGGTAG |
| Name | Sequence |
| EST11 | TTCTACGGGGCTGTGCTG |
| EST12 | AGATTGGTGGGGGTCGTG |
| EST13 | GTTTCTCAGCTTGGACTG |
| EST14 | TAGCATTGGGGATGTGAG |
| EST15 | AGCTCAGTAATGGACGAG |
| EST16 | GTCCGATCCATGTGAAAG |
| EST17 | AGCTTGGCTTATGAGGTG |
| EST18 | TACTCGTCCATACTTCGG |
| EST19 | CACTTCATGGGCAGTTTG |
| EST20 | CAGAGCGATTACGTCAAG |
| EST21 | TGGCCACCATTTGGCCAG |
| EST22 | ACCGTGCGAGTTACACGG |
| EST23 | ACGCGAGCCGAACAGAAG |
| EST24 | CATGGTCTGGTGACTCGG |
| EST25 | GCAACGTACATTGTCTGG |
| EST26 | GATGACGGACAAAGATCC |
| EST27 | TAAGTTCCTACCTGCGTG |
| EST28 | TTCTCTACTCAACGGCAC |
| EST29 | TCACGACAGCTCAATAGG |
| EST30 | AGTGTTTGAGCAGATCCC |
| EST31 | GTTGTCATGGTTGTTCCC |
| EST32 | GTCCAAGGTTGATTCGAG |
| EST33 | ACCGATTTTGCTACCCAC |
| EST34 | GATAACCAACATGCCCTC |
| EST35 | CTTCATCCAAACTGGTGG |
| EST36 | ACCAATAAGCCGTGAGAG |
| EST37 | GCCCCATAAGCCATTTAG |
| EST38 | CGTTCCTGACATGATAGG |
| EST39 | CACTAAAACTGGGCTCTC |
| EST40 | TGCCTGCTTATCTACTGG |
| EST41 | GACCAGACATTATGACGG |
| EST42 | CGTGGAACAATATGGCTC |
| EST43 | AAAAGAATGCCGGACGAG |
| EST44 | TGAGAAAGCGGATGTCTC |
| EST45 | GCGATAGAGTTAGTAGGG |
| EST46 | TTGCCCGATAGGTATCTC |
| EST47 | TGTGTTTCCGTTGACGAG |
| EST48 | CCAACCAGCATTTAGTCC |
| EST49 | GTACCTAATAGGGACCAC |
| EST50 | ATGAGACTTCCGGGATAG |
| EST51 | CAGAAACGACATAGCAGG |
| EST52 | TTGAGGCGAATGGTTCTG |
| EST53 | GGATTTGTCCGGTTCTAC |
| EST54 | AGTTCAAGTGTCATGGGG |
| EST55 | TTCCCCTGAGTATAGGAG |
| EST56 | GCCAGTCCAGTAATACAC |
| EST57 | CAAGCTTCCAAGCAACAG |
| EST58 | ACGGAAGCTCTGTACATC |
| EST59 | CGCAAATAGCGCCCACAG |
| EST60 | TTTGCAGTCACCGCCTCC |

Primer sequences used for *Biomphalaria glabrata* ORESTES libraries
