# Supplementary material for: Biomphalaria glabrata transcriptome: Identification of cell-signalling, transcriptional control and immune-related genes from open reading frame expressed sequence tags (ORESTES)
Source: Dev Comp Immunol. 2007;31(8):763–82. doi: 10.1016/j.dci.2006.11.004 (PMC1871615; doi:10.1016/j.dci.2006.11.004)
Supplement: Supplementary file 2 — Online Supplementary Materials [file mmc2.ppt]

## Slide 1
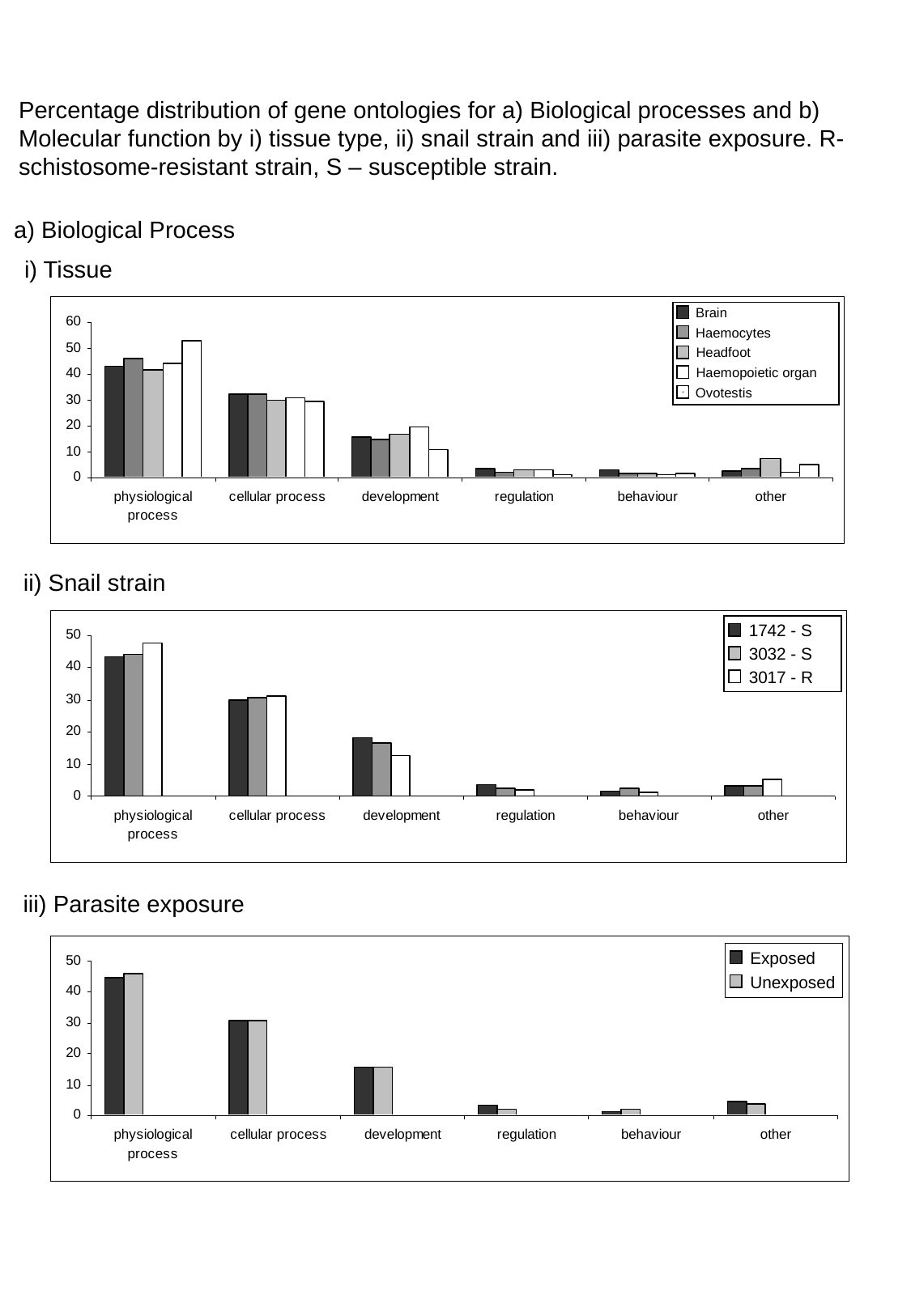

Percentage distribution of gene ontologies for a) Biological processes and b) Molecular function by i) tissue type, ii) snail strain and iii) parasite exposure. R- schistosome-resistant strain, S – susceptible strain.
a) Biological Process
i) Tissue
Brain
Haemocytes
Headfoot
Haemopoietic organ
Ovotestis
ii) Snail strain
1742 - S
3032 - S
3017 - R
iii) Parasite exposure
Exposed
Unexposed

## Slide 2
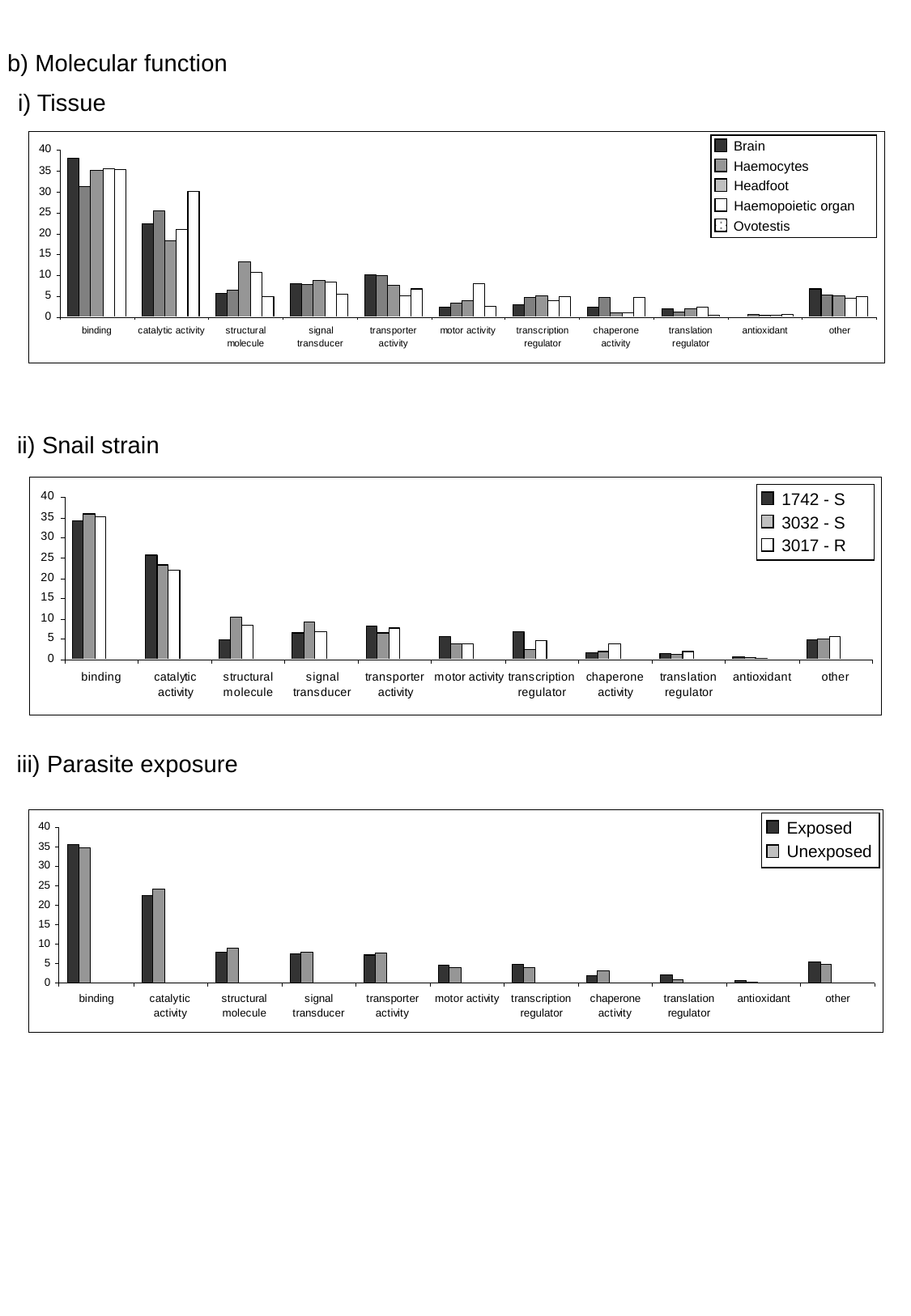

b) Molecular function
i) Tissue
Brain
Haemocytes
Headfoot
Haemopoietic organ
Ovotestis
ii) Snail strain
1742 - S
3032 - S
3017 - R
iii) Parasite exposure
Exposed
Unexposed
